# Editorial: Enhancing companion animal welfare through advanced behavioral management strategies

**DOI:** 10.3389/fnbeh.2026.1781387

**Published:** 2026-02-17

**Authors:** Yasemin Salgirli Demirbas, Kevin James McPeake, Joana Soares Pereira, Xavier De Jaeger

**Affiliations:** 1 Department of Psychology, Faculty of Arts, University of Prince Edward Island, Charlottetown, PE, Canada; 2 Department of Physiology, Ankara Universitesi Veteriner Fakultesi, Ankara, Türkiye; 3 The Royal (Dick) School of Veterinary Studies, University of Edinburgh, Edinburgh, United Kingdom; 4 Egas Moniz School of Health and Science, Campus Universitário, Instituto Universitario Egas Moniz Centro de Investigacao Interdisciplinar Egas Moniz, Caparica, Portugal; 5 Ceva Santé Animale, Libourne, France

**Keywords:** behavioral management, companion animal, human-animal bond, problem behavior, welfare

Behavioral problems in companion animals rarely have simple causes. They often emerge from complex interactions between species-specific behaviors, physical and social environments as well as the way caregivers interpret and respond to those behaviors. Developing and implementing effective management strategies therefore remains challenging, yet critically important, as failure to address these multifaceted contributors may pose a risk to animal welfare, ultimately weakening the human—animal bond.

Our Research Topic brings together four studies that explore different aspects of this challenge by adopting a comprehensive approach to companion animal behavioral management. In this Research Topic, Sonowal et al. compared scented and non-scented chew toys to evaluate their effect on chewing and mouthing behaviors in puppies, which are among the most common developmental issues faced by caregivers during critical bonding periods. Although these behaviors are largely normal communicatory and exploratory behaviors, they are frequently reported as problematic and can strain the human-animal bond at a sensitive stage of development. Authors showed that sensory enhanced toys significantly increased puppy engagement and reduced the need for caregiver prompting during observed sessions. Interestingly, while the frequency of unwanted chewing did not change over the course of the intervention, caregivers reported perceiving mouthing as less problematic. This finding highlights the importance of how caregivers experience and interpret behavior when managing behavioral problems in companion animals and offers a valuable insight for veterinary behavioral medicine regarding the key role of caregiver perspectives.

Distinguishing primary behavioral disorders from those arising secondary to medical conditions such as pain is one of the most challenging areas in behavioral medicine and was addressed by Kwik et al. within our Research Topic. Their case series revealed that maladaptive pain was present in 70% of dogs referred for behavioral complaints when assessed using a multimodal approach. This finding carries important implications for clinical practice and raises important questions such as: “*How often are animals diagnosed with behavioral problems before pain is thoroughly investigated?*” and “*How frequently do behavior modification efforts fail because an underlying pain remains untreated?*”. Their study demonstrates that multimodal treatment addressing both pain and behavior led to recovery in most cases, which highlights the inseparable relationship between medical and behavioral assessment. It further underscores the significant welfare consequences of diagnostic delay, resulting in unnecessary animal suffering and caregiver distress.

Feline behavioral management is the focus of two interesting studies in this Research Topic. In order to better understand undesired scratching in domestic cats, Demirbas et al. employed a multifactorial approach and identified the risk factors that contribute to this behavior. Their findings provide valuable insight into the environmental, social and individual factors that influence when normal feline behavior becomes problematic in the domestic setting. They identified stress as a leading contributor to undesired scratching, with specific risk factors including the presence of children in the household, high levels of play and nocturnal activity as well as personality traits such as aggression or disruptiveness. The authors emphasize that scratching, while a normal species-typical behavior, becomes problematic when underlying stressors are not adequately addressed.

This etiological perspective is complemented by the Endersby et al., who present a large-scale, controlled evaluation of a pheromone-impregnated collar for the management of multiple feline behavioral problems. With nearly 500 cats included, the findings provide robust evidence that continuous pheromone delivery can significantly reduce behavioral problems such as inappropriate urination, destructive scratching and inter-cat conflict, with trends toward improvement in fear-related behaviors. These results are particularly important, as these behaviors are among the most commonly reported problems in the companion cat population and represent predominant reasons for relinquishment and, in some cases, euthanasia. Their study highlights the value of pheromonal signals in reducing stress by promoting emotional stability in cats and demonstrates how effective pheromone messaging can be in managing a range of behavioral problems.

Collectively, the studies in this Research Topic highlight a number of fundamental concepts that deserve to be included into standard clinical practice. These include the critical importance of systematic pain assessment in cases presenting with behavioral change; recognition that caregiver perceptions and expectations may be influential determinants of treatment success and must be addressed; the value of identifying risk factors to inform management strategies and the role of sensory modulation as a foundational strategy for supporting emotional wellbeing of companion animals. This Research Topic supports the view that advancing the field requires moving beyond simplistic approaches toward integrative frameworks and adaptive expectations, with evidence-based strategies that enhance quality of life and strengthen the human-animal bond.

